# Recurrence of Animal Rabies, Greece, 2012

**DOI:** 10.3201/eid2002.130473

**Published:** 2014-02

**Authors:** Konstantia E. Tasioudi, Peristera Iliadou, Eirini I. Agianniotaki, Emmanuelle Robardet, Emmanouil Liandris, Spiridon Doudounakis, Mirsini Tzani, Paraskevi Tsaroucha, Evelyne Picard-Meyer, Florence Cliquet, Olga Mangana-Vougiouka

**Affiliations:** Ministry of Rural Development and Food, Athens, Greece (K.E. Tasioudi, P. Iliadou, E.I.Agianniotaki, E. Liandris, S. Doudounakis, M. Tzani, P. Tsaroucha, O. Mangana-Vougiouka);; WHO Collaborating Centre for Research and Management in Zoonoses Control, Malzéville, France (E. Robardet, E. Picard-Meyer, F. Cliquet);; OIE Reference Laboratory for Rabies, Malzéville (E. Robardet, E. Picard-Meyer, F. Cliquet);; European Union Reference Laboratory for Rabies, Malzéville (E. Robardet, E. Picard-Meyer, F. Cliquet);; European Union Reference Laboratory for Rabies Serology, Malzéville (E. Robardet, E. Picard-Meyer, F. Cliquet);; Technopôle Agricole et Vétérinaire, Malzéville (E. Robardet, E. Picard-Meyer, F. Cliquet)

**Keywords:** Rabies, Greece, rabies diagnostic, RT-PCR, molecular epidemiology, phylogenetic analysis, viruses, zoonoses, animal rabies

**To the Editor:** Rabies is caused by 12 recognized virus species within the *Lyssavirus* genus (family *Rhabdoviridae*) (*1*) and each year causes 55,000 deaths worldwide among humans. In Europe, the main reservoir and vector of rabies is the red fox (*Vulpes vulpes*), followed by the raccoon dog (*Nyctereutes procyonoides*) in central and Baltic Europe (*2*). Among these virus species, the rabies virus (former genotype 1, classical rabies virus) is maintained in reservoir mammals, mainly carnivores. Despite the successful oral vaccination campaigns of wildlife that eliminated rabies in large parts of Europe (*2*), the disease still occurs in Europe; 4,884 cases in animals were recorded in 2012.

Until 2012, Greece had been free of rabies since 1987; the last case occurred in a dog. During 1971–1987, a total of 248 cases were recorded in domestic animals, of which only 6 occurred during 1981–1987 (*3*). The wide compulsory vaccination of dogs, along with control of stray dogs, were the main measures leading to the elimination of rabies in Greece.

Greece shares land borders with Turkey, Former Yugoslav Republic of Macedonia (FYROM), and Bulgaria, where rabies is reported in wild and domestic animals (*4*; www.who-rabies-bulletin.org/). After the fox rabies case reported in FYROM in 2011, ≈0.3 km from Greece (*4*), rabies surveillance was improved along the northern and eastern land borders of Greece, and wild and domestic animals found dead or suspected of having rabies were collected. This program was approved by the European Commission. The first rabies case was diagnosed on October 19, 2012, in a red fox in Palaiokastro, 60 km from the Albanian border. The animal was wandering in the village during daytime and attacked a dog before being killed by local hunters and sent to the National Reference Laboratory for Animal Rabies (Athens, Greece) for rabies testing. The second case, isolated in Ieropigi, 4 km from the Albanian border, was a shepherd dog demonstrating aggressive behavior against dogs and sheep of the flock. Seven additional cases (6 foxes, 1 dog) were reported in December 2012 in 2 prefectures of northern Greece that share borders with FYROM (*5*).

In 2012, a total of 237 domestic and wild animals were submitted to the National Reference Laboratory for Animal Rabies for rabies testing. Samples were tested by the fluorescent antibody test, rabies tissue culture infection test, real-time reverse transcription PCR, and heminested reverse transcription PCR, as described (*6*,*7*). In brief, viral RNA was extracted (Viral RNA Mini Kit, QIAGEN, Hilden, Germany) from 140 μL of homogenized brain suspension supernatant and subjected to the partial nucleoprotein (N) gene amplification (positions 71–644 compared with PV strain genome). The PCR products were bidirectionally sequenced by using the same primers in a 3130xl Genetic Analyzer (Applied Biosystems, Foster City, CA, USA). The phylogenetic tree was constructed by using the neighbor-joining method with 1,000 replicates using MEGA5 (*8*).

Of 237 animals tested, 9 (7 foxes, 2 dogs) were rabies positive by fluorescent antibody test, rabies tissue culture infection test, and PCR. Positive samples were subjected to sequencing analysis of the N gene. The sequence analysis of the first 567 nt of the N gene of the 3 isolates (GR64C/12, GR112C/12, GR238C/12) showed 99.8% nt identity with the N gene of a rabies virus (GenBank accession no. JQ973884) isolated from a red fox in FYROM in 2011. Nucleotide identity was 100% between the 6 isolates from Greece (GR177C/12, GR187C/12, GR217C/12, GR231C/12, GR236C/12, GR242C/12) and isolate JQ973884, as well as a Serbia strain (GenBank accession no. JF973785). This perfect nucleotide identity shown with the Serbia isolate collected >15 years ago suggests the persistence of some viral strains over time in the Balkans, in accordance with previous studies (*4*,*9*). Amino acid identity was perfect among all 9 isolates from Greece.

Phylogenetic analysis of the partial sequences of the N gene of isolates from Greece compared with representative sequences from the Balkans (Figure) showed that isolates from Greece resolved within the East Europe (EE) group of the cosmopolitan lineage. The EE group encompasses the 9 Greek isolates with referenced viral sequences from FYROM, Bulgaria, Serbia, Bosnia Herzegovina, and Montenegro. Within the EE group, <1.5%-nt divergence exists between all analyzed N gene sequences (567 nt).

**Figure:**
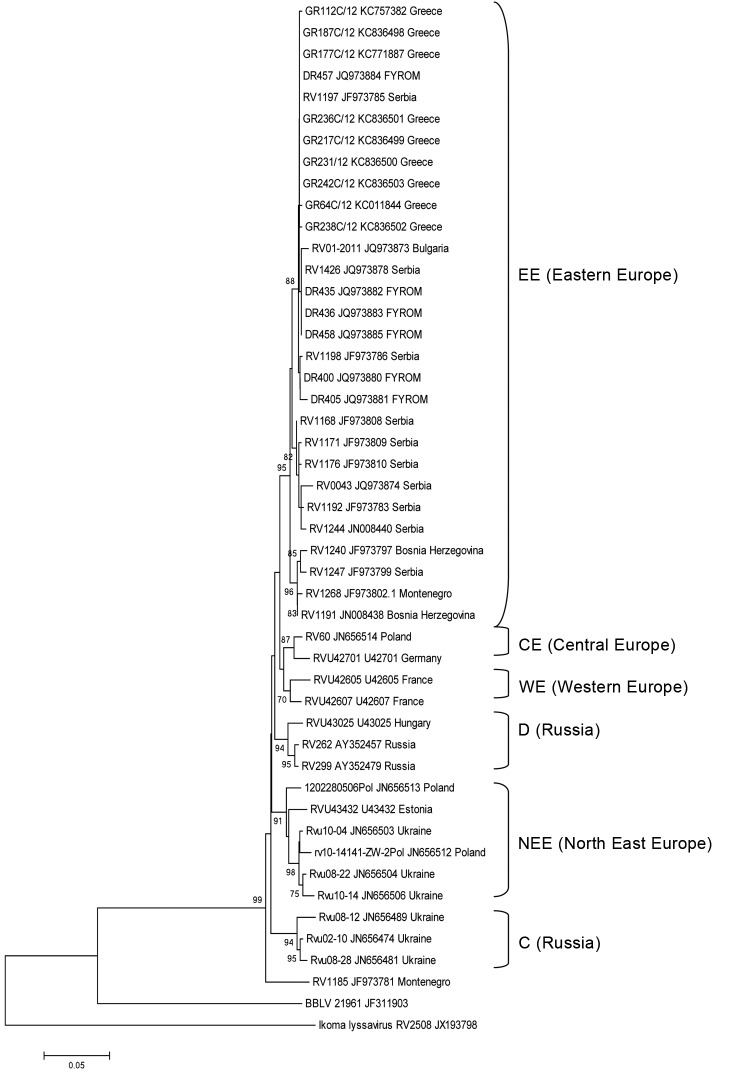
Neighbor-joining phylogenetic tree comparing 9 isolates (7 red foxes, 2 dogs) from Greece with isolates from Former Yugoslav Republic of Macedonia, Bulgaria, and Serbia. All 9 samples were isolated in Greece during October 19, 2012–December 28, 2012. Representative isolates from central, western, and northeastern Europe and from Russia, extracted from GenBank, also were included in the phylogenetic tree. The phylogenetic analysis was based on analysis of the first 567 nt of the N gene by using the neighbor-joining method (Kimura 2-parameter model). Bootstrap values >70% were regarded as significant support of tree topology. The GenBank accession numbers of the sequences are included for each taxon within the tree, as is the country of origin. Abbreviations for the phylogenetic groups (NEE, C, D, SF) are indicated on the tree. Scale bar indicates nucleotide substitutions per site.

The perfect nucleotide identity shown between the 6 isolates from Greece and 2 strains from Serbia and FYROM demonstrates a close genetic relationship between them. This finding supports the hypothesis of movement of rabies-infected hosts in the western Balkan countries.

Greece was rabies free for 25 years. In the 2012 outbreak, the rabies cycle appears to be sylvatic, whereas until 1987, dogs were the main reservoir of rabies. Measures including public awareness campaigns and intensified vaccination of stray animals and shepherd dogs, combined with control of stray dogs and cats, already have been implemented. In accordance with the current European Commission recommendations, Greece continued rabies surveillance and eradication program throughout the country in 2013, including the implementation of an oral rabies vaccination program for foxes in autumn 2013 (*10*).
